# Molecular Response to Toxic Diatom-Derived Aldehydes in the Sea Urchin *Paracentrotus lividus*

**DOI:** 10.3390/md12042089

**Published:** 2014-04-04

**Authors:** Stefano Varrella, Giovanna Romano, Adrianna Ianora, Matt G. Bentley, Nadia Ruocco, Maria Costantini

**Affiliations:** 1 Stazione Zoologica Anton Dohrn, Villa Comunale, Naples 80121, Italy; E-Mails: stefano.varrella@szn.it (S.V.); giovanna.romano@szn.it (G.R.); adrianna.ianora@szn.it (A.I.); nadia.ruocco @szn.it (N.R.); 2 Dove Marine Laboratory, School of Marine Science and Technology, Newcastle University, Newcastle upon Tyne, UK; E-Mail: matt.bentley@newcastle.ac.uk

**Keywords:** aldehydes, molecular targets, sea urchin, teratogenesis

## Abstract

Diatoms are dominant photosynthetic organisms in the world’s oceans and represent a major food source for zooplankton and benthic filter-feeders. However, their beneficial role in sustaining marine food webs has been challenged after the discovery that they produce secondary metabolites, such as polyunsaturated aldehydes (PUAs), which negatively affect the reproductive success of many invertebrates. Here, we report the effects of two common diatom PUAs, heptadienal and octadienal, which have never been tested before at the molecular level, using the sea urchin, *Paracentrotus lividus*, as a model organism. We show that both PUAs are able to induce teratogenesis (*i.e.*, malformations), as already reported for decadienal, the better-studied PUA of this group. Moreover, post-recovery experiments show that embryos can recover after treatment with all three PUAs, indicating that negative effects depend both on PUA concentrations and the exposure time of the embryos to these metabolites. We also identify the time range during which PUAs exert the greatest effect on sea urchin embryogenesis. Finally, we report the expression levels of thirty one genes (having a key role in a broad range of functional responses, such as stress, development, differentiation, skeletogenesis and detoxification processes) in order to identify the common targets affected by PUAs and their correlation with morphological abnormalities. This study opens new perspectives for understanding how marine organisms afford protection from environmental toxicants through an integrated network of genes.

## 1. Introduction

Diatoms are a highly productive class of microalgae, widespread in both marine and freshwater habitats, that are widely fed upon by both planktonic and benthic invertebrates. However, while diatoms may provide a source of energy for larval growth, they often reduce fecundity and/or hatching success or cause malformations (teratogenesis) during growth, due to the production of secondary metabolites, such as polyunsaturated aldehydes (PUAs) and other products deriving from the oxidation of polyunsaturated fatty acids (PUFAs), collectively termed oxylipins (reviewed by [1] and [2]). This biological model is new and has no other equivalent in marine plant-herbivore systems, since most of the known negative plant-animal interactions are generally related to repellent or poisoning processes, but never to reproductive failure. 

Oxylipins, and PUAs in particular, have important biological and biochemical properties, including the disruption of gametogenesis, gamete functionality, fertilization, embryonic mitosis and larval fitness and competence [1]. The dominant bioactive PUAs released by diatoms are C_10_ 2-*trans*-4-*trans*-decadienal, 2-*trans*-4-*cis*-7-*cis*-decatrienal and 2-*trans*-4-*trans*-7-*cis*-decatrienal [3], but also C_8_ 2-*trans*-4-*cis*-7-octatrienal, 2-*trans*-4-*trans*-7-octatrienal, 2-*trans*-4-*cis*-7-octadienal, 2-*trans*-4-*trans*-7-octadienal, C_7_ 2-*trans*-4-*cis*-7-heptadienal and 2-*trans*-4-*trans*-7-heptadienal [4,5]. The first two PUAs were isolated from the freshwater diatom, *Melosira varians*, by Wendel and Jüttner [6], but the biological activity of these molecules was not known at the time. Miralto *et al.* [3] showed, for the first time, that they arrested the embryonic development of copepod and sea urchin embryos in a dose-dependent manner and also had anti-proliferative and apoptotic effects on human carcinoma cells. Successive studies have shown that sea urchin gametes incubated with decadienal have impaired fertilization success, due to the inhibition of sperm motility [7,8]. At concentrations higher than the dose required to arrest cell cleavage progression, decadienal induced apoptotic events in *Paracentrotus lividus* embryos by inducing caspase-3-like protease activity [9].

Hansen *et al.* [10] studied the effects of decadienal on the sea urchin, *Sphaerechinus granularis*, and showed that this PUA inhibited cyclin B/Cdk1 kinase activity, DNA replication and tubulin polymerization, leading to arrest of the cell cycle. Romano *et al.* [11] showed that PUAs compromised embryonic and larval development of sea urchins even at low doses and that the most deleterious of the PUAs tested were the longer chain aldehydes, such as decadienal.

The first molecular studies on the effects of PUAs on the sea urchin, *P. lividus*, were reported very recently [12,13]. In particular, newly fertilized sea urchin eggs were exposed to low concentrations of decadienal, and the expression levels of seventeen genes, implicated in a broad range of functional responses, were followed by real-time qPCR. At low decadienal concentrations, the sea urchin, *P. lividus*, activated different classes of genes to defend itself against this toxic aldehyde, ranging from canonical stress genes to developmental and skeletogenic genes [13]. The authors suggested that this orchestrated defence system against decadienal represents part of the chemical defensome of *P. lividus*, affording protection from environmental toxicants.

Since information on the molecular effects of PUAs are scant and mostly related to the effects induced by decadienal, the aim of the present study was to explore the effects of two other ecologically important aldehydes, heptadienal and octadienal, which have never been tested before on *P. lividus* embryos from the molecular point of view, and to compare these effects with those induced by decadienal. The sea urchin, *P. lividus*, is considered a good model system to study the ecotoxicological response of marine invertebrates to environmental pollutants for several reasons: its ecological relevance, benthic and relatively sedimentary lifestyle, rapid response and high sensitivity to many types of contaminants, transparent embryos that grow rapidly in the laboratory and its long reproductive period. For these reasons, we decided to adopt it for our study and treated sea urchin embryos with increasing concentrations of heptadienal and octadienal to analyse morphological changes induced by exposure to these natural products and to define their mechanism of action and possible teratogenic activity. We also followed, by real-time qPCR, thirty one genes to identify potential target genes. Fourteen of these genes, having a key role in a broad range of functional responses, such as development, differentiation and detoxification processes, are now compared to those investigated in our previous study on the effects of decadienal on *P. lividus* development [13]. 

## 2. Results

### 2.1. Determination of Teratogenic Effects and PUA Dose-Dependent Concentrations

As reported in previous studies [11,12], decadienal induced teratogenesis at low concentrations, with an increase in the number of abnormal plutei in the sea urchin, *P. lividus*. Marrone *et al.* [13] also reported a dose-dependent effect of this PUA in *P. lividus*, with severe malformations in plutei, such as asymmetrical arms and spicules and reduced arm length and shortening of the apex. Romano *et al.* [12] showed that a decadienal concentration of 1.6 μM (0.25 μg/mL) was ideal to study morphogenetic changes in embryo development and gene expression levels in *P. lividus*, with the production of about 35% abnormal plutei.

**Figure 1:**
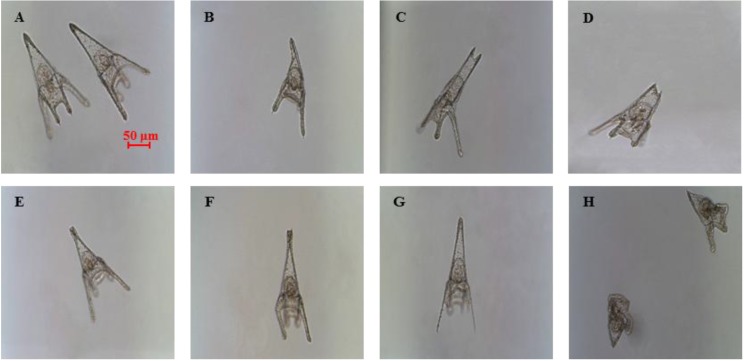
Examples of malformations induced in *Paracentrotus lividus* plutei at 48 hours post fertilization (hpf) after incubation with the three polyunsaturated aldehydes (PUAs), heptadienal, octadienal and decadienal (**B**–**H**) in comparison with (**A**), the control; embryos are in sea water without aldehydes.

The present study explores further the effects of decadienal at the molecular level and compares these effects with those induced by two other PUAs, heptadienal and octadienal, which have never been tested before on *P. lividus* embryo development. Treatment with the three PUAs induced similar malformations in the plutei (see Figure 1), with effects on the arms, spicules and apex. Such plutei had a poorly-formed apex (Figure 1B) because of the spicules that appeared either parallel or disjoined at the tip (Figure 1C,D) or crossed at the apex (Figure 1E), with arms that appeared longer and broader or crooked and asymmetrical (Figure 1F) or completely degenerated (Figure 1G), compared to controls (Figure 1A). At times, the entire body plan of the plutei was strongly compromised and malformed (Figure 1H). For a more detailed overview of the abnormal plutei produced by PUAs, see Supplementary Figure S1.

**Figure 2:**
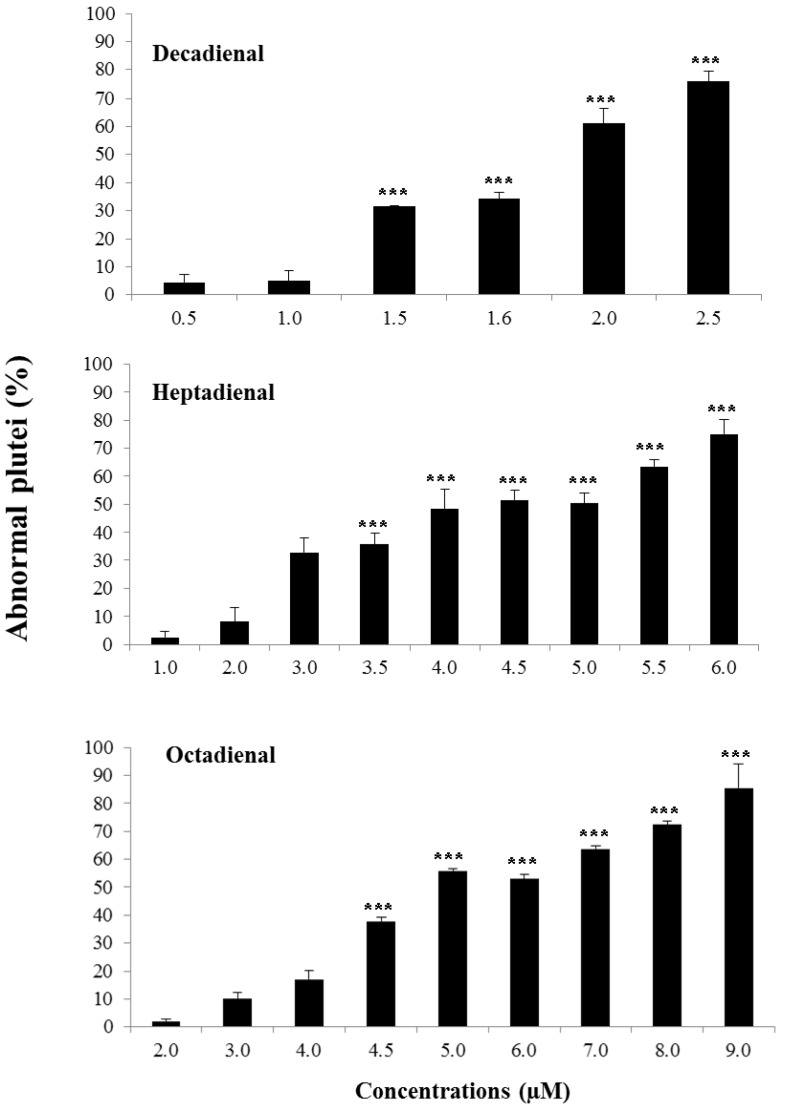
Percentage of abnormal plutei (%) produced when *Paracentrotus lividus* newly-fertilized eggs are exposed to different concentrations (in μM) of heptadienal, octadienal and decadienal for 48 h post fertilization (*** with a *p*-value <0.001, Student’s *t*-test, GraphPad Software Inc.,San Diego, CA, USA).

The percentage of abnormal plutei was calculated at the different concentrations tested (Figure 2; see also the Experimental Section for more details). 

The effect was dose-dependent for the three PUAs tested, even if the range of concentrations inducing teratogenesis differed (from 0.5 to 2.5 μM for decadienal, from 1.0 to 6.0 μM for heptadienal and from 2.0 to 9.0 μM for octadienal). Decadienal was the strongest of the three, because of the very narrow range (2.0 μM) that affected embryonic development; heptadienal and octadienal required higher ranges of concentrations to reach the same effects as decadienal. Therefore, we chose as teratogenic concentrations 3.0 μM for heptadienal and 4.5 μM for octadienal, in order to obtain the same percentage of abnormal plutei (about 35%), as in the case of decadienal. Controls were also performed in filtered sea water (FSW) and in FSW plus methanol, and we found that methanol had no interference with the embryo development. In fact, the percentage of abnormal plutei was the same for embryos in FSW, as well as in FSW plus methanol. 

### 2.2. Post-Recovery Experiments

**Figure 3:**
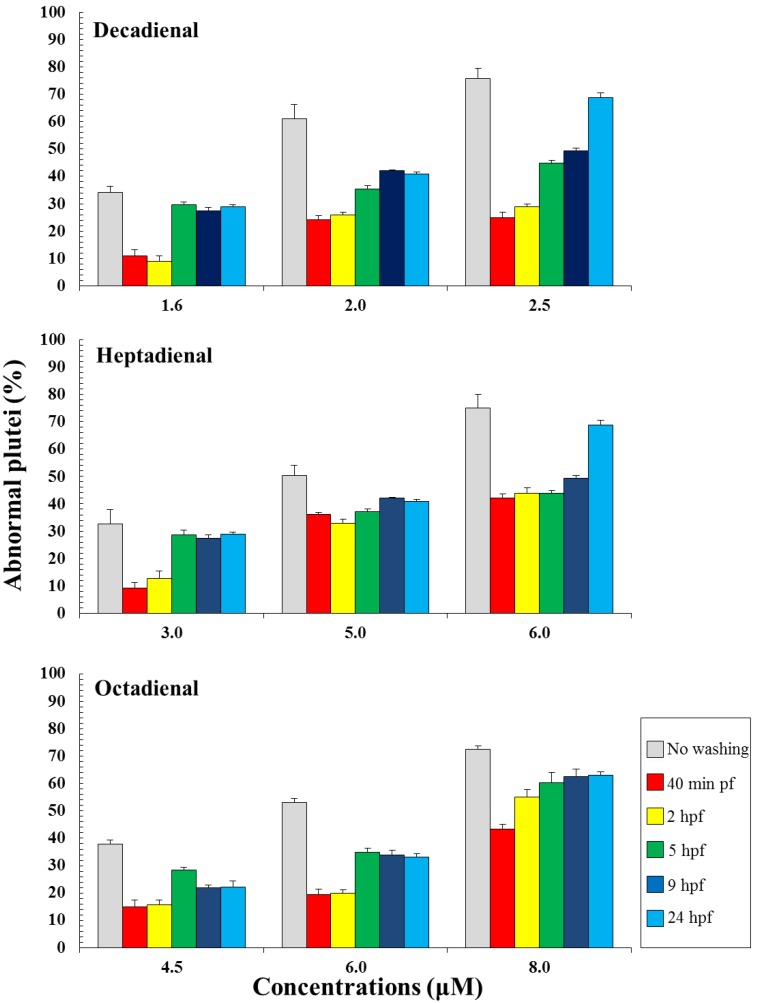
Percentage of abnormal *Paracentrotus lividus* plutei (%) produced after exposure to different concentrations of heptadienal, octadienal and decadienal and at different development times after fertilization. The percentages of abnormal plutei without washing are also reported.

Post-recovery experiments were performed in order to investigate if sea urchin embryos were able to recover after exposure to these PUAs. Eggs were incubated with three different concentrations of PUAs and then fertilized. The lowest concentrations represented the concentration inducing teratogenesis for each PUA (decadienal 1.6 μM, heptadienal 3.0 μM, octadienal 4.5 μM) and were also used to study the gene stress response (see below); the other concentrations (decadienal two and 2.5 μM; heptadienal five and 6 μM; octadienal six and 8 μM) were chosen to have comparable percentages of abnormal plutei (about 60% and 75%). Embryos were washed at different times after fertilization, 40 min, two, five, nine and 24 hours post fertilization (hpf), corresponding to two-cell, eight-cell, early blastula, swimming blastula and prism, respectively. After washing, embryos were grown to the pluteus stage to calculate the number of abnormal embryos. The results indicate that embryos were able to recover at all concentrations tested and at all times after fertilization (Figure 3), with the exception of the highest concentrations at 24 hpf. Moreover, the results suggest that octadienal could have a different mechanism of action compared to decadienal and heptadienal, because embryos were less able to recover at all times after fertilization at the highest concentrations. According to these results, we conclude that the post-recovery effects depend both on PUA concentrations and the exposure time of embryos to these metabolites, with the exception of octadienal at the highest concentration.

### 2.3. Addition of PUAs at Different Developmental Stages

**Figure 4:**
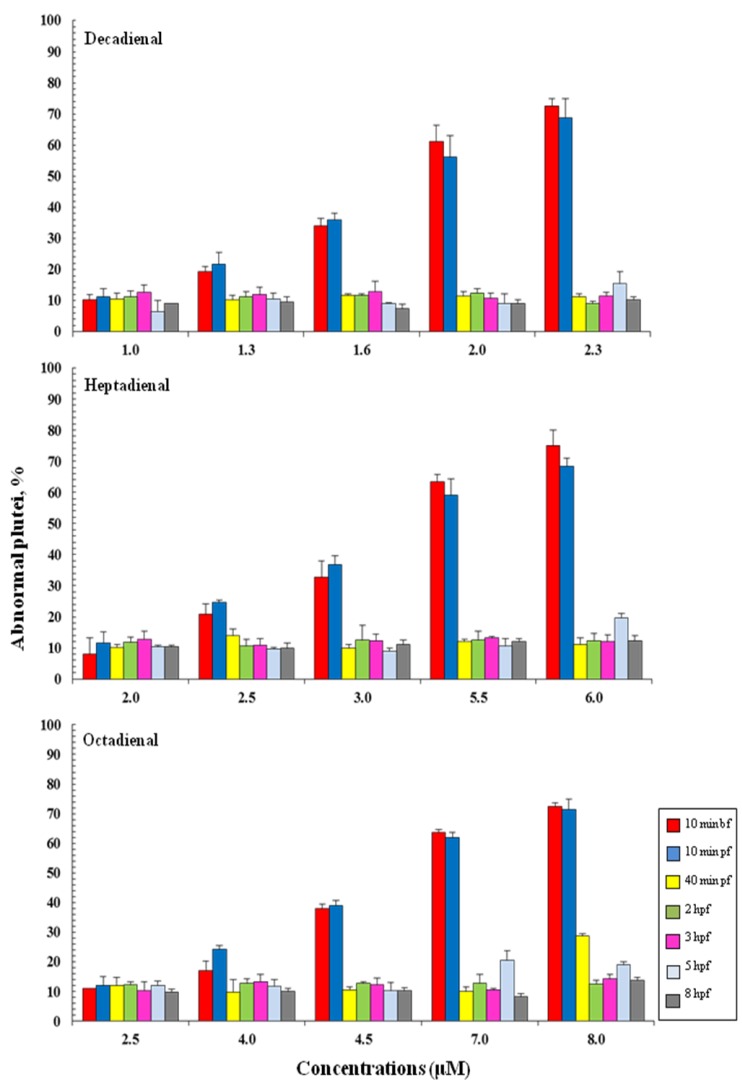
The percentage of abnormal *Paracentrotus lividus* plutei (%) produced after the exposure of newly fertilized eggs to different concentrations of heptadienal, octadienal and decadienal and examined at different developmental stages: 10 min before fertilization (bf), 10 min and 40 min post fertilization (pf) and two, three, five and 8 hpf.

To define the stage at which PUAs affect embryo development, aldehydes were added: 10 min before fertilization (bf), 10 min pf, 40 min pf, 2, 3, 5 and 8 hpf. These stages represented key stages during sea urchin embryogenesis. In fact, 40 min pf corresponds to the two-cell stage; from this stage until 5 hpf, only mitotic cell division occurs; at 5 hpf (corresponding to early blastula stage) the differentiation of blastomeres begins, and cell fates in specific embryonic territories are defined; at 8 hpf, the embryo envelope starts to be digested, so the embryos comes into direct contact with the external environment for the first time. The results show that the addition of PUAs affect embryonic development in the same way, whether they are added 10 min before fertilization (bf) and/or 10 min pf, resulting in the same percentage of abnormal plutei (Figure 4). The addition of PUAs in later developmental stages does not seem to affect the embryonic development of *P. lividus*. In fact, the percentage of abnormal plutei remains very low (about 10%–20%) for each concentration tested. 

### 2.4. Gene Stress Response to PUAs

To better understand the morphological effects at the molecular level, *P. lividus* embryos were allowed to develop in the presence of PUAs at concentrations inducing teratogenesis (1.6 μM for decadienal, 3.0 μM for heptadienal and 4.5 μM for octadienal), and embryos were collected at different development times after fertilization, corresponding to the stages of early blastula (5 hpf), swimming blastula (9 hpf), prism (24 hpf) and pluteus (48 hpf). The expression levels of thirty one genes (see the Experimental Section for more details) were followed by real-time qPCR to identify potential gene targets. These genes are implicated in a broad range of functional responses, such as stress, development, differentiation, skeletogenesis and detoxification processes (see Table 1). 

**Table 1:** The function for the genes analysed in the present study.

Gene Name	Acronym	Function	Reference
			
***Pl-p19***	*p19*	small acidic proteins, involved in the formation of the	[14]
***Pl-p16***	*p16*	biomineralised skeleton of sea urchin embryos and adults	
			
***ALG-2 interacting protein X/1***	*Alix*	protein involved in endocytic membrane trafficking,	[15]
		filamentous (F)-actin remodelling and cytokinesis	
			
***Blimp***	*Blimp*	zinc finger transcription factor, which plays a central role	[16]
		in both early and late endomesoderm specification	
			
***Wnt 5***	*Wnt5*	initiates the specification of the sea urchin posterior ectoderm	[17]
***Wnt 6***	*Wnt6*	activates endoderm in the sea urchin gene regulatory network	[18]
***Wnt 8***	*Wnt8*	endomesodermal specification, embryo patterning, early	[19]
		primary mesenchyme cells-gene regulatory network regulator	
			
***Multi drug resistance protein 1***	*MDR1*	ATP-binding cassette protein	[20]
			
***Metallothionein 4***	*MT4*		
***Metallothionein 5***	*MT5*	proteins capable of binding to heavy metals, involved in	[21]
***Metallothionein 6***	*MT6*	the transport of heavy metals and cellular detoxification	
***Metallothionein 7***	*MT7*		
***Metallothionein 8***	*MT8*		
			
***Catalase***	*Cat*	antioxidant defensive protein	

**Figure 5:**
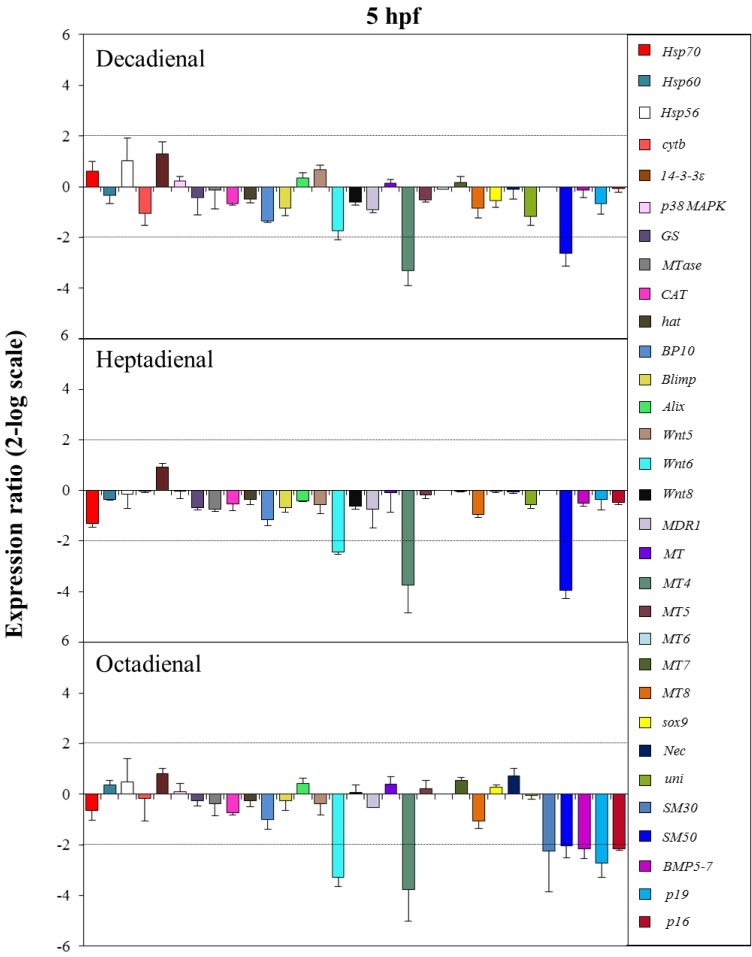
The histograms show the differences in the expression levels of thirty one genes followed by real-time qPCR. *Paracentrotus lividus* embryos were grown in the presence of decadienal, heptadienal and octadienal at teratogenic concentrations (1.6, 3.0 and 4.5 μM, respectively) and collected at 5 hpf. Data are reported as a fold difference (mean ± SD), compared to the control embryos in sea water without aldehydes. Fold differences greater than ±2 (see the dotted horizontal guide lines at the values of +2 and −2) were considered significant.

**Figure 6:**
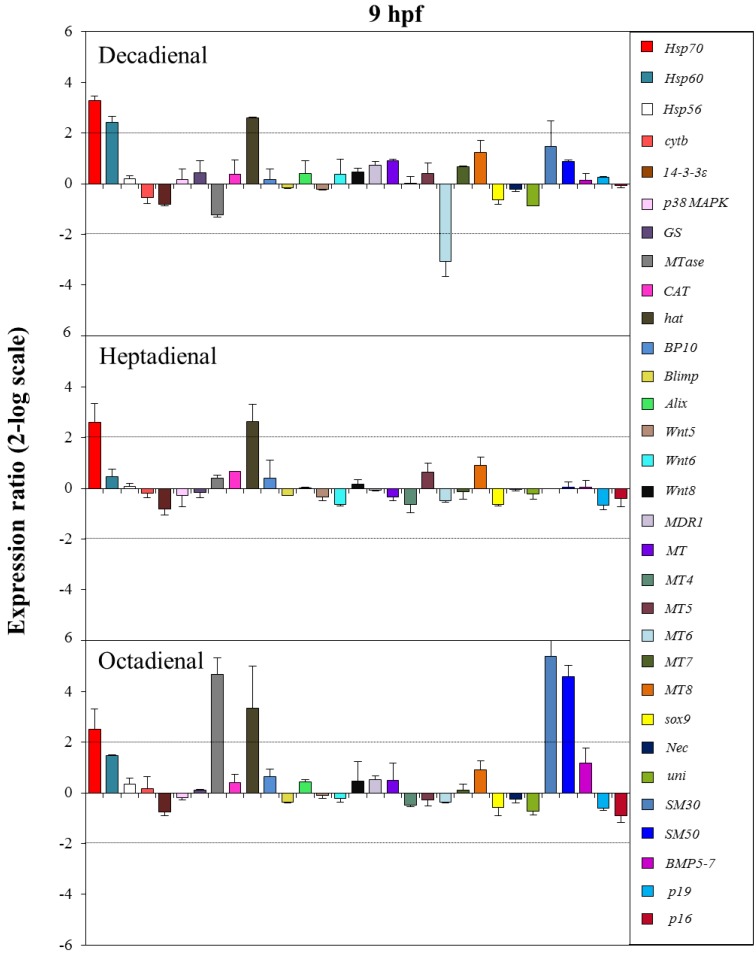
The histograms show the differences in the expression levels of thirty one genes followed by real-time qPCR. *Paracentrotus lividus* embryos were grown in the presence of decadienal, heptadienal and octadienal at teratogenic concentrations (1.6, 3.0 and 4.5 μM, respectively) and collected at 9 hpf. For more details, see also the legend to Figure 5.

**Figure 7:**
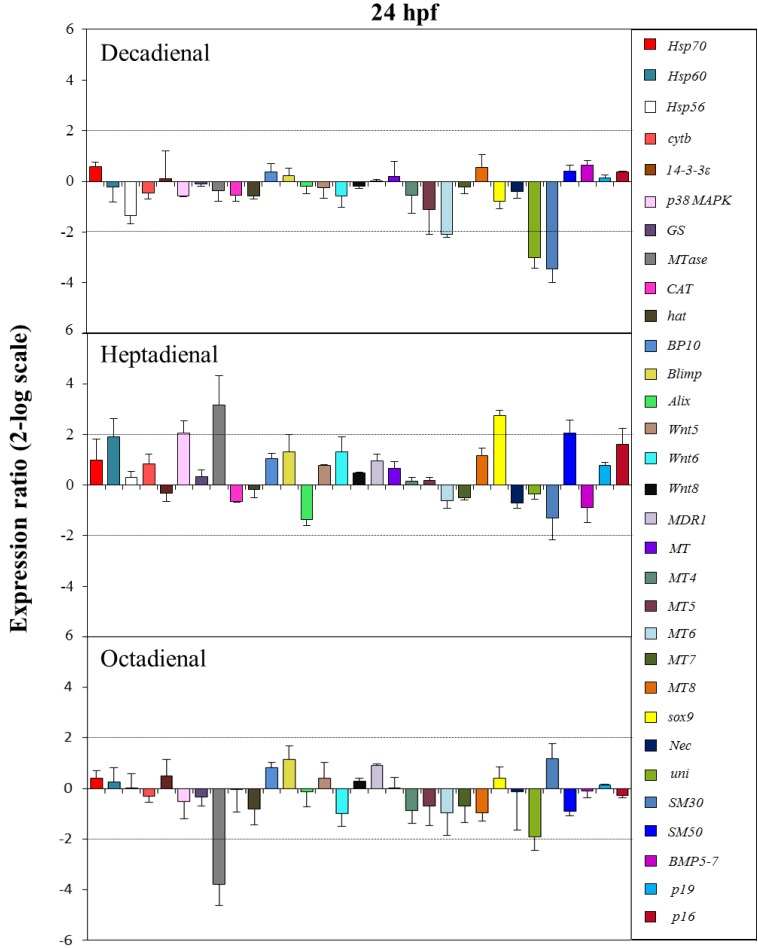
The histograms show the differences in the expression levels of thirty one genes followed by real-time qPCR. *Paracentrotus lividus* embryos were grown in the presence of decadienal, heptadienal and octadienal at teratogenic concentrations (1.6, 3.0 and 4.5 μM, respectively) and collected at 24 hpf. For more details, see also the legend to Figure 5.

**Figure 8:**
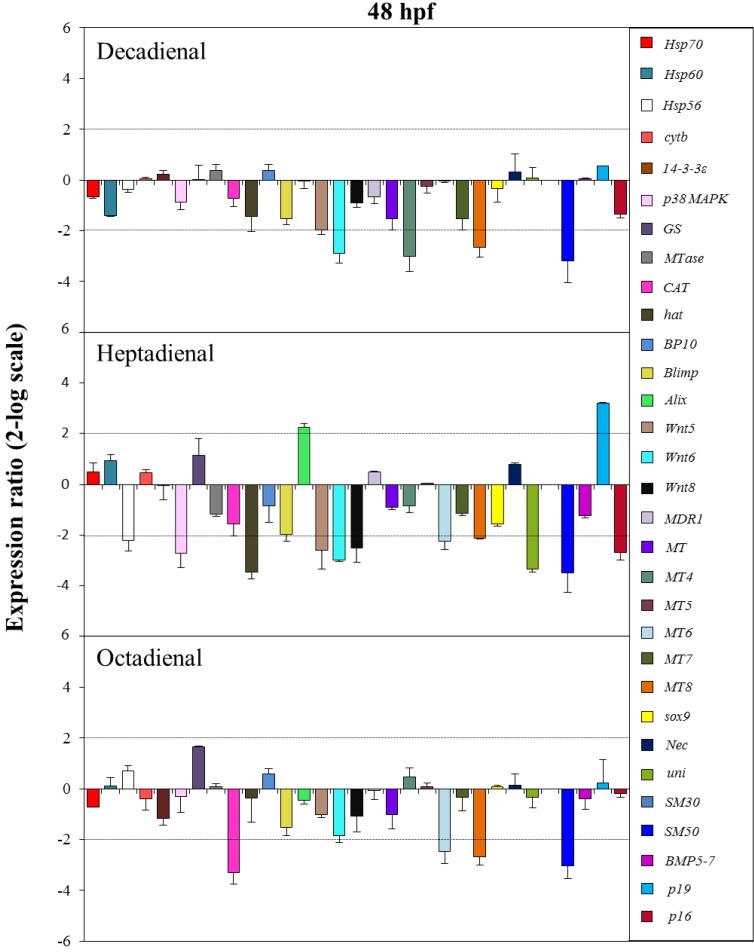
The histograms show the differences in the expression levels of thirty one genes followed by real-time qPCR. *Paracentrotus lividus* embryos were grown in the presence of decadienal, heptadienal and octadienal at teratogenic concentrations (1.6, 3.0 and 4.5 μM, respectively) and collected at 48 hpf. For more details, see also the legend to Figure 5.

The control gene for real-time qPCR was ubiquitin, the expression of which remained constant in all sea urchin developmental stages. The histograms reported in Figure 5, Figure 6, Figure 7 and Figure 8 show the relative expression ratios of the analysed genes with respect to control embryos in sea water without PUAs. Only expression values greater than a two-fold difference with respect to the controls were considered significant. 

At the early blastula stage (5 hpf; see Figure 5), one metallothionein and one skeletogenic gene, *MT4* and *SM50*, were targeted by all three PUAs, showing a 2.6-, 4.0- and 2.1-fold decrease in expression levels with respect to the control, respectively. The expression level of *Wnt6*, a gene implicated in the developmental and differentiation processes, was significantly reduced by heptadienal (2.4-fold) and octadienal (3.3-fold) compared to the control. Moreover, at this stage, only octadienal affected the expression levels of four skeletogenic genes, which were downregulated: *SM30*, *Bmp5-7*, *p19* and *p16*, showing a 2.2-, 2.2-, 2.7- and 2.2-fold decrease with respect to the control, respectively.

At the swimming blastula stage (9 hpf; see Figure 6), two genes were upregulated: the stress gene *hsp70* (3.2- for decadienal, 2.6- for heptadienal and 2.5-fold for octadienal) and the protease *hat* (2.6-fold for decadienal and heptadienal; 3.5-fold for octadienal). Moreover, treatment with decadienal showed a 2.4-fold increase in the expression level of the stress gene, *hsp60*, and a three-fold decrease for metallothionein *MT6*. At this stage, octadienal activated one stress gene and two skeletogenic genes: *MTase*, *SM30* and *SM50*, with a 4.7-, 5.4- and 4.6-fold increase with respect to the control.

At the prism stage (24 hpf; see Figure 7), heptadienal and octadienal differentially affected the expression levels of the stress gene, *MTase*. Whereas heptadienal upregulated this gene with a 3.2-fold increase, octadienal downregulated with a 3.8-decrease in expression levels. At this developmental stage, the genes targeted by heptadienal were the stress gene, *p38 MAPK*, and the skeletogenic gene, *SM50*, both of which showed a 2.1-fold increase in expression levels with respect to the control, and the developmental gene, *sox9*, with a 2.7-fold increase. Decadienal affected the expression levels of the metallothionein, *MT6*, and the two skeletogenic genes, *uni* and *SM30*, with a 2.1-, 3- and 3.5-fold downregulation, respectively.

At the pluteus stage (48 hpf; see Figure 8), all three PUAs targeted the metallothionein, *MT8*, and the skeletogenic *SM50* genes. In particular, the expression levels of these genes were all downregulated: *MT8* had a 2.7-fold decrease for decadienal and octadienal and a 2.1-fold decrease for heptadienal; *SM50* a 3.2-, 3.5- and 3.1-fold decrease for decadienal, heptadienal and octadienal, respectively. The metallothionein *MT6* gene was targeted by both heptadienal and octadienal, with a 2.2- and 2.5-fold downregulation of their expression levels, respectively. Decadienal and heptadienal induced a 2.9-fold downregulation of *Wnt6*. Moreover, decadienal also affected the expression level of the metallothionein *MT4* gene, causing a 3-fold decrease with respect to the control. Heptadienal upregulated the expression level of two genes, the developmental gene, *Alix* (2.2-fold), and the skeletogenic gene, *p19* (3.2-fold), and downregulated several genes: two stress genes, *hsp56* and *p38 MAPK* (2.2- and 2.7-fold decrease, respectively); three developmental genes, *hat, Wnt5* and *Wnt 8* (3.5, 2.6- and 2.5-fold decrease, respectively); and two skeletogenic genes, *uni* and *p16* (3.4 and 2.7-fold decrease, respectively). Octadienal was the only PUA that downregulated the *CAT* gene (3.3-fold decrease) implicated in detoxification processes. 

## 3. Discussion

Our results greatly expand on previous investigations on the stress response to the toxic PUA, decadienal, during sea urchin development [12,13]. Here, we probe more deeply into the effects induced by two ecologically important, but relatively unknown, PUAs, heptadienal and octadienal, which have never been tested before on *P. lividus* embryos from the molecular point of view, in comparison with the better-known PUA, decadienal. Since mainly heptadienal and octadienal are released when diatom cells are wounded during grazing [5,22,23] or lysed from senescent cells during bloom periods [24], it should be interesting to determine the direct effects of these pure molecules. Until now, very few studies have tested the effects of pure PUAs on copepods [25,26,27] or reported their effects at the molecular level in copepods [28,29] or the sea urchin, *P. lividus* [12,13]. Sea urchins have been widely used as a sensible bioindicator of biochemical, morphological and physiological changes related to environmental stressors, such as pesticides, essential and heavy metals, ionizing radiations, ocean warming and acidification, metal nanoparticles and natural toxins [30,31,32,33,34,35,36,37,38,39].

**Figure 9:**
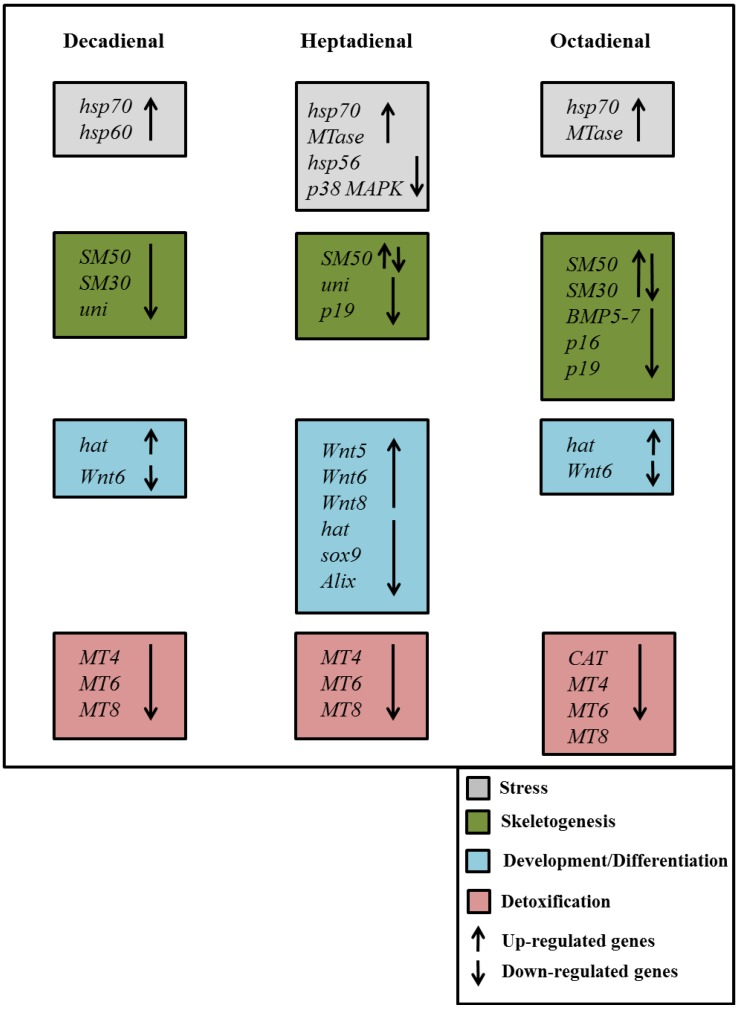
Synopsis of the patterns of up- and down-regulation of different classes of genes in the sea urchin, *Paracentrotus lividus*, in the presence of the PUAs decadienal, heptadienal and octadienal.

An important outcome of this study was the finding that the expression levels of a great number of genes in the sea urchin, *P. lividus*, appears to be modulated by the PUAs, decadienal, heptadienal and octadienal. A synopsis showing the patterns of up- and down-regulation of different classes of genes is shown in Figure 9. These three aldehydes have very few common targets, but specifically affect different classes of genes and at different times of development. 

Of the stress genes, the canonical stress gene, *hsp70*, was affected by the three aldehydes at the same development time (at the swimming blastula stage), confirming that embryos were subjected to stress, resulting in the activation of this gene as a first defence system [12]. The other two heat shock proteins, *hsp60* and *hsp56*, were also targeted by PUAs; these genes are reported as a part of a protection system against different stressors enhancing cell survival and normal cellular homeostasis [12,28,40,41,42]. 

The possible role of DNA methylation as a molecular marker in response to stress [43] was confirmed by the increase in the expression level of *MTase* after treatment with heptadienal at the prism stage and octadienal at the swimming blastula and prism stages. The expression level of *p38 MAPK*, participating in a signalling cascade in response to different stimuli [33], was affected by heptadienal at the prism and pluteus stages. Very recently, Pinsino *et al.* [44] emphasized the role of p38 MAPK in the regulation of sea urchin embryonic development after exposure to manganese.

Of the genes involved in development and differentiation processes, PUAs affected the expression levels of *hat*, *sox9*, *alix* and *Wnt*. We found an increase in the expression level of *hat*, an early embryonic messenger transiently expressed during the blastula stage [45,46] with all three PUAs. The *sox9* gene was upregulated by heptadienal at the prism stage, suggesting an effect on left-right asymmetry processes [47]. *Alix*, a multifunctional protein involved in different cellular processes, including endocytic membrane trafficking, filamentous-actin remodelling and cytokinesis [15], was upregulated only by heptadienal at the pluteus stage. In sea urchins, this transcript encodes for a maternal protein involved in determination/differentiation events that is expressed from fertilization to the two-cell embryo stage. In sea urchin eggs, the gene is localized throughout the cytoplasm with a punctuated pattern, and soon after fertilization, it accumulates in the cytosol and in microvilli-like protrusions. 

Of the genes belonging to the canonical Wnt pathway, *Wnt6* was targeted by all three PUAs that induced a decrease in the expression levels of this gene. This represents a very interesting result, supporting the essential role of Wnt6 to in triggering endoderm specification [18]. In fact, heptadienal and octadienal affected the expression level of this gene at 5 hpf, when the differentiation of blastomeres occurs and cell fates in specific embryonic territories are defined. Heptadienal was also able to determine a decrease in the expression levels of two other genes of the Wnt pathway: *Wnt5*, which acts as a short-range inter-blastomere signal, activating cells in the ectoderm, integrating different types of positional information from both the primary and secondary embryonic axes in order to correctly locate the production site of signals needed for skeleton formation to take place [17]; and *Wnt8* is associated with cell fate determination through canonical signalling pathways and is important for the morphogenetic movement of primary mesenchyme cells [19].

All genes involved in skeletogenesis were downregulated by PUAs, except for the *Nec* gene. *SM30*, *SM50* and *Uni* were previously found to be target genes for decadienal [13]. Octadienal downregulated the expression level of *BMP5-7*, a gene family that is reported as being positive regulators of oral and aboral ectoderm specification [17]. *P19* was upregulated by heptadienal, but was downregulated by octadienal together with *p16*; these are two small acidic proteins involved in the formation of the biomineralised skeleton of sea urchin embryos and adults [14]. 

We also analysed eight genes involved in detoxification processes. In particular, of six metallothioneins that were analysed in this work, only the expression levels of three genes appeared to be perturbed by PUAs. Ragusa *et al.* [21] reported that two metallothionein genes (*MT7* and *MT8*) appeared to be constitutively expressed and upregulated upon cadmium treatment, whereas other genes (*MT4*, *MT5* and *MT6*) were not transcribed in control embryos and were specifically activated in response to cadmium treatment. Our results show the down-expression of the induced metallothioneins, *MT4* and *MT6*, and the constitutive one, *MT8*, at a later stage of development (48 hpf). The induction of other metallothioneins may require exposure to higher concentrations of PUAs or may depend on the nature of the stress agent, considering that metallothioneins seem to respond very specifically to heavy metal exposure. The gene, *MDR1*, belongs to ATP-binding cassette transporters, which are activated by sub-lethal doses of specific contaminants (such as oxybenzone, mercuric chloride and tributyltin) during embryonic development (from the zygote to the blastula stage) of sea urchins [20]. Moreover, sea urchin embryos utilize it in cell signalling and lysosomal and mainly mitochondrial homeostasis [48]. The expression level of this gene was not affected by PUA exposure, probably due to the low levels of exposure. In fact, we demonstrated the loss of mitochondrial functionality only at higher concentrations of decadienal in a previous study [12]. The down-expression of the *CAT* gene only in the case of octadienal suggests that a specific detoxification system could be activated in sea urchins after exposure to this PUA.

These molecular results are in accordance with our morphological results that revealed that the majority of malformations affected the skeleton and the plan of the development and differentiation of sea urchin embryos, as reported in Figure 1. In fact, several genes belonging to the skeletogenic, developmental and differentiation classes were affected by PUAs. Even if, according to our morphological analyses, octadienal seems to be the weakest of the three aldehydes, our study on gene expression levels reveals that this aldehyde affects a very early stage of embryonic development. In fact, octadienal differentially affected the expression levels of five skeletogenic genes at the early blastula stage compared to the other aldehydes. These data are also in accordance with our post-recovery experiments, in which we hypothesized that octadienal could have a different mechanism of action, because embryos cannot recover in the presence of this aldehyde at the highest concentration. This different behaviour could be correlated with the downregulation of skeletogenic genes at an early stage of embryo development, which does not give embryos the chance to recover. The strongest molecular effects seem to occur after treatment with heptadienal, but at a later stage of development. In fact, at 48 hpf (pluteus stage), heptadienal affected the expression levels of thirteen genes. Taken together, these results suggest that although treatment with the three aldehydes did not induce any visible differences at the morphological level, they affected different physiological processes.

The genes identified in this work as targets for PUAs may represent possible biomarkers to detect exposure to pollutants that may include microbial products, heavy metals and phytotoxins. More generally, the new genes analysed in this work can be considered as an additional part of the stress surveillance system or chemical defensome of the sea urchin, as proposed in Marrone *et al.* [13], affording protection from environmental xenobiotics.

## 4. Experimental Section

### 4.1. Ethics Statement

*Paracentrotus lividus* (Lamarck) sea urchins were collected from a location that is not privately-owned or protected in any way, according to Italian legislation of the Marina Mercantile (Decreto del Presidente della Repubblica DPR 1639/68, 09/19/1980 confirmed on 01/10/2000). The field studies did not involve endangered or protected species. All animal procedures were in compliance with the guidelines of the European Union (Directive 609/86).

### 4.2. Gamete Collection, Embryo Culture, Exposure to Aldehydes and Morphological Analysis

Adult sea urchins of the species, *P. lividus*, were collected during the breeding season by scuba-diving in the Gulf of Naples, transported in an insulated box to the laboratory within 1 h after collection and maintained in tanks with circulating sea water until testing. Sea urchins were injected with 2 M KCl through the peribuccal membrane to obtain the emission of gametes. Eggs were washed with filtered sea water (FSW) and kept in FSW until use. Concentrated sperm was collected, dried and kept undiluted at +4 °C until use.

Before fertilization, eggs were incubated at room temperature for 10 min in the presence of different concentrations of the three PUAs: 2*-trans*,4-*trans-*decadienal at 1.0, 1.3, 1.6, 2.0, 2.3 μM (similar to the concentrations tested in reference [13]); 2-*trans*,4-*trans-*heptadienal at 1.0, 2.0, 3.0, 3.5, 4.0, 4.5, 5.0, 5.5, 6.0 μM; 2-*trans*,4-*trans*-octadienal (Sigma-Aldrich, Milan, Italy) at 2.0, 3.0, 4.0, 4.5, 5.0, 6.0, 7.0, 8.0, 9.0 μM; and the controls were in FSW without PUAs.

Eggs were fertilized utilising sperm-to-egg ratios of 100:1 for both controls and treated embryos. Fertilized eggs were kept at 20 °C in a controlled temperature chamber on a 12 h:12 h light:dark cycle. PUAs were diluted in methanol, considering a methanol to FSW ratio of 10 μL:1 mL, so as to avoid interference with embryo development. Controls were also performed in FSW and in FSW in the presence of methanol. 

Experiments were conducted in triplicate using three egg groups collected from three different females. After 48 h of incubation, morphological malformations were determined for at least 200 plutei using a light microscope (Zeiss Axiovert 135TV, Carl Zeiss, Jena, Germany) in comparison to control embryos in FSW without PUAs.

### 4.3. Post-Recovery Experiments

The procedure for the treatments with the three PUAs was the same as reported above. For each PUA, three concentrations were tested according to the percentage of abnormal plutei recorded during previous tests. The lowest concentrations were those inducing teratogenesis: 1.6 μM for decadienal, 3.0 μM for heptadienal and 4.5 μM for octadienal, all three of which induced the production of about 35% abnormal plutei that were also used for molecular experiments (see below). We then tested the concentrations that induced the production of about 60% and 75% of abnormal plutei, so as to have comparable results with the three PUAs. Embryos were washed twice at different development times: 40 min and 2, 5, 9 and 24 h post-fertilization (hpf). Embryos were grown to the pluteus stage. The number of abnormal embryos was evaluated by fixing embryos in formaldehyde (4% in FSW) and counting under the light microscope.

One-way ANOVA with Tukey’s post-hoc test was performed using GraphPad Prism version 4.00 for Windows (GraphPad Software, San Diego, CA, USA).

### 4.4. Addition of PUAs after Fertilization

Eggs were fertilized without PUAs, according to the procedure reported above. The development of embryos was followed by microscopic examination for different development times after fertilization (10 min pf, 40 min pf, 2, 3, 5 and 8 hpf). PUAs were then added at the same concentrations used for post-recovery experiments. As controls, PUAs were also added 10 min before fertilization. The number of abnormal plutei was evaluated from fixed embryos. One-way ANOVA with Tukey’s post-hoc test was performed using GraphPad Prism version 4.00 for Windows (GraphPad Software, San Diego, CA, USA).

### 4.5. RNA Extraction and cDNA Synthesis

About 30,000 eggs in 200 mL of FSW were treated for 10 min with the three PUAs at the following concentrations: 1.6 μM for decadienal (as in [13]); 3.0 μM for heptadienal; and 4.5 μM for octadienal. Eggs were then fertilized and collected at different developmental times. Samples were collected at 5, 9, 24 and 48 hpf by centrifugation at 1800 relative centrifugal force for 10 min in a swing out rotor at 4 °C. The pellet was washed with phosphate buffered saline and then frozen in liquid nitrogen and kept at −80 °C. 

Total RNA was extracted from each developmental stage using TRIzol (Invitrogen, Life Technologies, Carlsbad, CA, USA) according to the manufacturer’s instructions. Extraction with chloroform/isoamyl alcohol (24:1) was performed following RNA precipitation by addition of glycogen and isopropyl alcohol. Contaminating DNA was degraded by treating each sample with a DNase RNase-free kit (Roche, Milan, Italy) according to the manufacturer’s instructions. The amount of total RNA extracted was estimated by the absorbance at 260 nm and the purity by 260/280 and 260/230 nm ratios, by a NanoDrop spectrophotometer (ND-1000 UV-Vis Spectrophotometer; NanoDrop Technologies, Wilmington, DE, USA). The integrity of RNA was evaluated by agarose gel electrophoresis. Intact rRNA subunits (28S and 18S) were observed on the gel indicating minimal degradation of the RNA. For each sample, 600 ng of total RNA extracted was retrotranscribed with an iScript™ cDNA Synthesis kit (Bio-Rad, Milan, Italy), following the manufacturer’s instructions. Synthetized cDNA was used in real-time qPCR experiments without dilution.

To evaluate the efficiency of cDNA synthesis, a PCR was performed with primers of the reference gene, ubiquitin. The reaction was carried out on the C1000 Touch Thermal Cycler GeneAmp PCR System 9700 (Applied Biosystem, Monza, Italy) in a 30 μL final volume with 3 μL 10× PCR reaction buffer (Roche, Milan, Italy), 3 μL 10× 2 mM dNTP, 1 μL 5 U/μL Taq (Roche, Milan, Italy), 100 ng/μL of each oligo, template cDNA and nuclease free water to 30 μL. The PCR program consisted of a denaturation step at 95 °C for 5 min, 35 cycles at 95 °C for 45 s, 60 °C for 1 min and 72 °C for 30 s and a final extension step at 72 °C for 10 min.

### 4.6. Gene Expression by Real-Time qPCR

For all real-time qPCR experiments, the data from each cDNA sample were normalized using ubiquitin mRNA as the endogenous control level, the level of which remained relatively constant in all developmental stages examined according to Nemer *et al.* ([49]; for more details, see Romano *et al.* [12]). The expression level of seventeen genes, previously analysed by real-time qPCR in Marrone *et al.* [13], were analysed together with fourteen new genes (see Table 1): Pl-p19 (*p19*), Pl-p16 (*p16*; [14]), metallothionein 4 (*MT4*; [21]), metallothionein 5 (*MT5*; [21]), metallothionein 6 (*MT6*; [21]), metallothionein 7 (*MT7*; [21], metallothionein 8 (*MT8*; [21]), blimp (*Blimp*; [16]), ALG-2 interacting protein X/1(*Alix*; [15]), multi-drug resistance protein 1 (*MDR 1*), Wnt5 (*Wnt5*), Wnt6 (*Wnt6*) and Wnt8 (*Wnt8*). We also analysed the catalase gene (*CAT*); since the *CAT* sequence of *P. lividus* is not available, a 156-bp fragment was amplified using specific primers for this gene from *Strongylocentrotus purpuratus*. The amplified fragment using a Taq High Fidelity PCR System (Roche, Milan, Italy) was purified from agarose gel using the QIAquick Gel Extraction kit (Qiagen, Milan, Italy), and the specificity of the PCR product for catalase was checked by DNA sequencing.

Gene sequences were retrieved from NCBI [50]. For each gene, specific primers were designed on the basis of nucleotide sequences of *P. lividus* (see Table 2); primers reported in the indicated references were used for six genes. The same procedure for the amplified fragments used for *CAT* was applied for these genes to check for the specificity of the amplified products.

**Table 2:** Gene name, acronym, accession numbers, primer sequences and lengths of PCR amplified fragments are reported for the genes analysed. For the genes that have a reference, the lengths of PCR fragments were not reported.

Gene Name	Acronym	Accession Number	Primer	Sequence (5′→3′)	PCR Fragment (bp)
					
***Pl-p19***	*p19*	FR693764	Pl_P19_F1	GACAAGCTCGACATCAACAAG	205
			Pl_P19_R1	CTGGAGTCGATGCTGCATCATG	
					
***Pl-p16***	*p16*	FR693763	Pl-p16 For	CGGGCAGCGATGACTCA	104
[14]			Pl-p16 Rev	AAATGCCATACCGCTCTTCTGT	
					
***Metallothionein 4***	*MT4*		MT4 For	GCTCAAAATCTTCAACATGGCTAATGA	
[21]			MT4 Rev	AGCACTTTCCAGTTTCACAACAAGC	
					
***Metallothionein 5***	*MT5*		MT5 For	CGACTTTAGCTCAAATTCATCACCATG	
[21]			MT5 Rev	TCCACAGCATTTACCATCCTTGC	
					
***Metallothionein 6***	*MT6*		MT6 For	CACGATTTGTGCTCAATCCTTCAT	
[21]			MT6 Rev	TTTGTGCATGATGTTCCACAGC	
					
***Metallothionein 7***	*MT7*		MT7 For	CGTCAAGAGATCAAAATCATCAACCA	
[21]			MT7 Rev	ACAGCACTCGCCAGTAATACAGCAC	
					
***Metallothionein 8***	*MT8*		MT8 For	GATGGTTGTCGTCGCTCCTAACA	
[21]			MT8 Rev	TCAAGAAAGGCTGGTATCAAATCTGAC	
					
***Blimp***	*Blimp*	HQ322503	Pl_Blimp1_For	CTGTCTACTCCATGCCGTCC	161
			Pl_Blimp1_Rev	GCCTCCTGCTTCAGATCAGC	
					
***ALG-2 interacting protein X/1***	*Alix*	HE646599	AL1500for	TACCAGACCATTCTCAACAAT	110
[15]			AL1610rev	TGCTATTTCCGCTTCGCTTTT	
					
***Multi drug resistance protein 1***	*MDR1*	JQ793791	Pl_MDR1_F2	GTCAAGGTACTCAATGGGGTC	158
			Pl_MDR1_Rev	CGGATGTCAATGCCATCAATC	
					
***Wnt 5***	*Wnt5*	HM449806	Pl_Wnt5_F2	CACCCAGCCCCTGTGCAGTG	135
			Pl_Wnt5_Rev	CTGCAGTTCCACCTCCTATTC	
					
***Wnt 6***	*Wnt6*	HQ322504	Pl_Wnt6_For	CGAATCTGCCGACGATCACG	164
			Pl_Wnt6_For	GCATTGTCGTACAGTTCCACC	
					
***Wnt 8***	*Wnt8*	HM449816	Pl_Wnt8_For	CTGTAAGTGTCATGGCGTCTC	197
			Pl_Wnt8_For	GAGCGAATCGGAGATGACGG	
					
***Catalase***	*CAT*	SPU_000281.1	Sp-CAT_F1	GACTTCGTCTTCACCGACGAG	156
			Sp-CAT_R1	GACTCAAAGGGTGCAGCCTTG	

The specificity of amplification reactions was verified by melting curve analysis. The efficiency of each primer pair was calculated according to standard method curves using the equation E=10^−1/slope^. Five serial dilutions were set up to determine Ct values and reaction efficiencies for all primer pairs. Standard curves were generated for each oligonucleotide pair using Ct values *versus* the logarithm of each dilution factor. PCR efficiencies were calculated for control and target genes and were found to be 2. Diluted cDNA was used as a template in a reaction containing a final concentration of 0.3 mM for each primer and 1× FastStart SYBR Green master mix (total volume of 10 μL) (Applied Biosystems, Monza, Italy). PCR amplifications were performed in a ViiATM7 Real Time PCR System (Applied Biosystems, Monza, Italy) thermal cycler using the following thermal profile: 95 °C for 10 min, one cycle for cDNA denaturation; 95 °C for 15 s and 60 °C for 1 min, 40 cycles for amplification; 72 °C for 5 min, one cycle for final elongation; one cycle for melting curve analysis (from 60 °C to 95 °C) to verify the presence of a single product. Each assay included a no-template control for each primer pair. To capture intra-assay variability, all real-time qPCR reactions were carried out in triplicate. Fluorescence was measured using ViiATM7 software (Applied Biosystems, Monza, Italy). The expression of each gene was analysed and internally normalized against ubiquitin using REST software (Relative Expression Software Tool, Weihenstephan, Germany) based on the Pfaffl method [51,52]. Relative expression ratios above two cycles were considered significant. Experiments were repeated at least twice. Statistical analysis was performed using GraphPad Prism version 4.00 for Windows (GraphPad Software, San Diego, CA, USA).

## 5. Conclusions

In conclusion, our findings provide molecular evidence for the toxic effects of diatom-derived PUAs and propose novel tools for understanding the cellular mechanisms of the response to aldehyde exposure in benthic organisms. Sea urchins may come in contact with diatom PUAs in the field at the end of a bloom, with the mass sinking of diatoms to the sediment, which represents a major source of organic matter for benthic systems [53]. This is of considerable ecological relevance given the importance of diatom blooms in nutrient-rich aquatic environments. Moreover, our results may be useful in understanding how changes in gene expression levels may be used as an early indicator of stressful conditions in the marine environment. Generally, these studies are fundamental to our understanding of how marine organisms attempt to defend themselves from environmental toxicants, benefitting from the protection provided by an integrated network of genes.

## Supplementary Files

Supplementary File 1 Supplementary Information (PDF, 123 KB)

## Abbreviations

FSW: filtered sea water
hpf: hours post fertilization
µL: microliter
µM: micromolar
min: minutes
mM: millimolar
nm: nanometer
