# Supplementary material for: Molecular Response to Toxic Diatom-Derived Aldehydes in the Sea Urchin Paracentrotus lividus
Source: Mar Drugs. 2014 Apr 4;12(4):2089–113. doi: 10.3390/md12042089 (PMC4012444; doi:10.3390/md12042089)
Supplement: Supplementary File 1 — Supplementary Information (PDF, 123 KB) [file marinedrugs-12-02089-s001.pdf]

Supplementary Information

Figure S1. Overview of the abnormal plutei after treatments with PUAs.

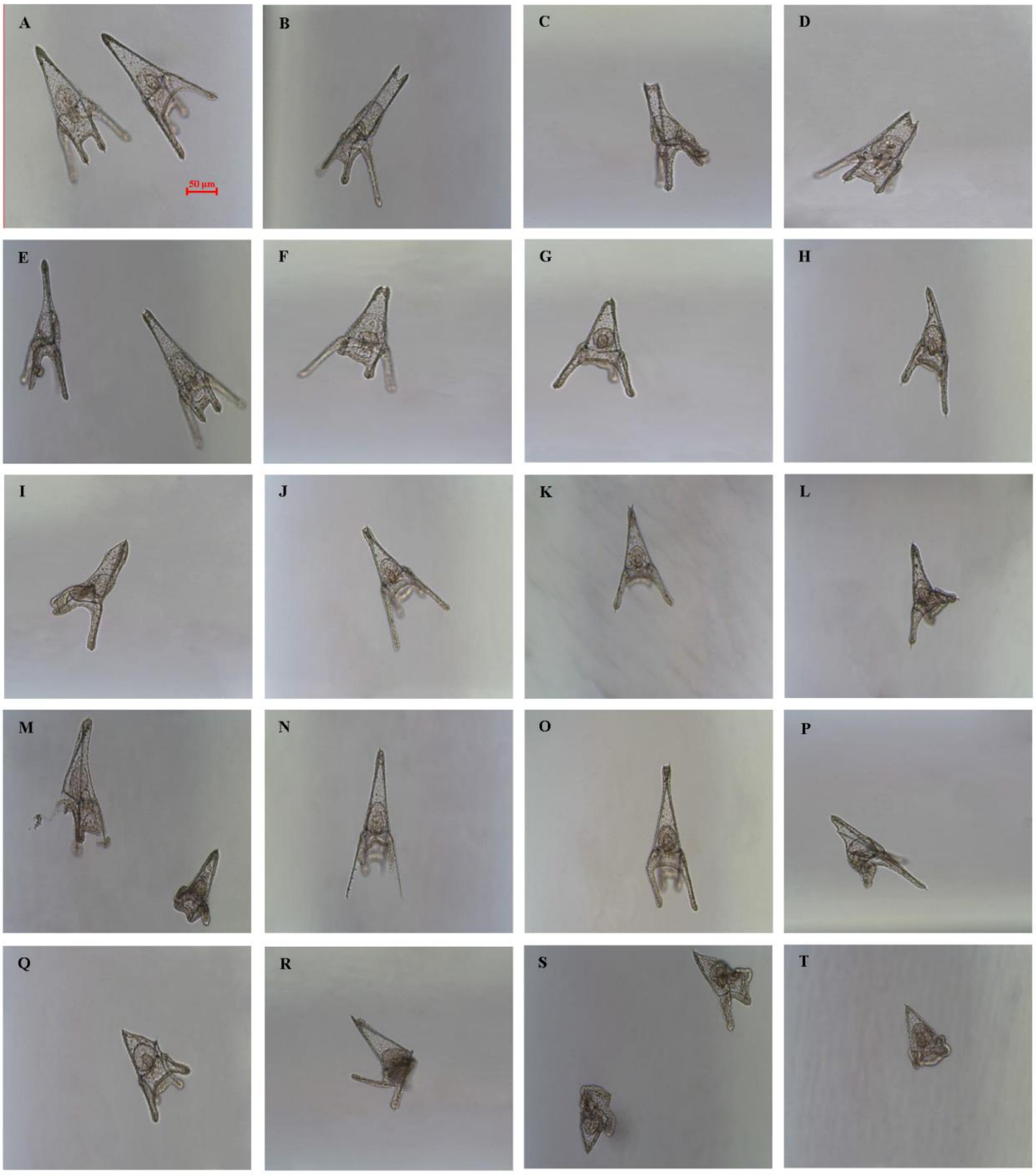

© 2014 by the authors; licensee MDPI, Basel, Switzerland. This article is an open access article distributed under the terms and conditions of the Creative Commons Attribution license (http://creativecommons.org/licenses/by/3.0/).
